# Effects of cannabis on eyewitness memory: A field study

**DOI:** 10.1002/acp.3414

**Published:** 2018-04-19

**Authors:** Annelies Vredeveldt, Steve D. Charman, Aukje den Blanken, Maren Hooydonk

**Affiliations:** ^1^ Department of Criminal Law and Criminology, Faculty of Law Vrije Universiteit Amsterdam Amsterdam The Netherlands; ^2^ Department of Psychology Florida International University Miami USA

**Keywords:** cannabis, drugs, eyewitness memory, lineup identification

## Abstract

Eyewitnesses to crimes are regularly under the influence of drugs, such as cannabis. Yet there is very little research on how the use of cannabis affects eyewitness memory. In the present study, we assessed the effects of cannabis on eyewitness recall and lineup identification performance in a field setting. One hundred twenty visitors of coffee shops in Amsterdam viewed a videotaped criminal event, were interviewed about the event, and viewed a target‐present or target‐absent lineup. Witnesses under the influence of cannabis remembered significantly fewer correct details about the witnessed event than did sober witnesses, with no difference in incorrect recall. Cannabis use was not significantly associated with lineup identification performance, but intoxicated witnesses were significantly better at judging whether their lineup identification was accurate. Theoretical and practical implications of the findings are discussed.

## 1 INTRODUCTION

Information provided by eyewitnesses is instrumental in solving criminal cases. Because eyewitness evidence is so important, a large body of research has investigated the many potential factors that may affect eyewitness memory. One such factor is witness intoxication during the crime. Eyewitnesses are frequently under the influence of alcohol and/or drugs while witnessing a crime (Evans, Compo, & Russano, 2009; Palmer, Flowe, Takarangi, & Humphries, 2013). Although a reasonable number of studies have assessed the effect of the most prevalent form of intoxication, alcohol inebriation, on eyewitness memory (see, e.g., Crossland, Kneller, & Wilcock, 2016; Hagsand, Roos af Hjelmsäter, Granhag, Fahlke, & Söderpalm Gordh, 2017; La Rooy, Nicol, & Terry, 2013; Schreiber Compo et al., 2017), research on other types of drugs in eyewitness settings is rare. The present paper describes a field experiment investigating the influence of cannabis intoxication on eyewitness statements about a criminal incident and lineup identification.

Law enforcement officers in the United States estimate that approximately 18% of witnesses are under the influence of marijuana, whereas 24% are under the influence of multiple substances (Evans et al., 2009). Archival analysis of criminal cases in the United States suggests that around 9% of witnesses had used cannabis or a combination of cannabis and alcohol (Palmer et al., 2013). The present study was conducted in the Netherlands, where figures on the prevalence of cannabis intoxication among eyewitnesses are not available. However, we do know that smoking cannabis is highly prevalent in the Dutch nightlife scene, with between 30% and 48% of people going out to a party, club, or bar reporting having used cannabis that night (WODC & Trimbos Instituut, 2016). Because criminal incidents are also likely to happen in the nightlife scene (Loef, Heijke, & Van Dijk, 2010), we can safely assume that many eyewitnesses are under the influence of cannabis when they witness a crime.

Cannabis intoxication affects memory. This is clear from a range of studies examining recall and recognition of simple stimuli such as word lists (e.g., D'Souza et al., 2004; Miller, Cornett, & Wikler, 1979; Miller, McFarland, Cornett, & Brightwell, 1977). In these studies, participants come to the laboratory and either use cannabis (experimental group) or not (control group). Participants are then presented with a set of items and asked to recall those items, either immediately or after a delay (or both). In most studies, participants also perform a recognition test after the recall phase. The findings consistently show that intoxicated participants recall fewer studied items than do sober participants, regardless of delay. It seems that the detrimental effect of cannabis intoxication operates at all stages of the memory process—encoding, consolidation, and retrieval—although findings are somewhat mixed as to at which stage cannabis causes the greatest detriments (see Ranganathan & D'Souza, 2006, for a review). Further, the decrease in correct recall is typically dose dependent: Greater doses of cannabis are associated with greater impairments in recall (D'Souza et al., 2004; Heishman, Arasteh, & Stitzer, 1997; Miller & Cornett, 1978). In contrast, cannabis intoxication is generally not associated with reduced correct recognition rates (e.g., Hart et al., 2010; Miller et al., 1977; Miller & Cornett, 1978). Thus, whereas cannabis makes it more difficult to retrieve information from memory, it does not seem to hinder the recognition of previously seen items.

Cannabis is associated not only with a decrease in correct recall but also with an increase in incorrect recall. Intoxicated participants are more likely to recall items that were never presented to them, that is, they are more likely to have false memories (Ranganathan & D'Souza, 2006). According to Ranganathan and D'Souza, this may be due to increased mental activity as a result of cannabis intoxication, leading to irrelevant associations. Although the effect of cannabis intoxication seems to be less pronounced for recognition tasks, some studies also show an increase in incorrect claims that a newly presented item has been seen before (i.e., false alarms; e.g., Hart et al., 2010; Ilan, Smith, & Gevins, 2004; Miller et al., 1977). Thus, in both recall and recognition tasks, cannabis intoxication can result in false memories.

The effect of cannabis on false memories was examined in more detail by Riba et al. (2015), who studied the effects of long‐term cannabis use rather than current intoxication. Participants in their experimental group had used cannabis daily for at least the last 2 years but were sober during the experiment. Participants studied a word list and then performed a recognition test consisting of old words, semantically unrelated new words, and semantically related new words (lures). Compared with a matched control group, heavy cannabis users did not differ in terms of correct recognition of old words or correct rejection of unrelated new words but were significantly more likely to falsely recognize lure words. Riba et al. explained their findings using a combination of fuzzy trace theory (Brainerd & Reyna, 2002), which holds that false memories occur when people rely on gist rather than verbatim memory, and the activation‐monitoring account (Balota et al., 1999), which holds that false memories occur when cognitive control mechanisms are not appropriately activated. Riba et al.'s neuroimaging data revealed that during the recognition test, regular cannabis users displayed less activation than did control participants both in brain regions associated with verbatim memory and in regions associated with gist memory. The authors argued that control participants engaged in concurrent retrieval of verbatim‐ and gist‐based memories, resulting in a conflict that prompted greater engagement of cognitive control mechanisms (as reflected in increased frontal activation), which helped control participants distinguish between old and new words. Regular cannabis users, in contrast, found it more difficult to determine that the lure words had not been presented during the learning phase. Thus, regular cannabis use can have lasting effects on cognitive mechanisms, rendering cannabis users more susceptible to false memories.

It is not clear to what extent the above‐mentioned findings from the basic memory literature extend to the type of complex memory task faced by eyewitnesses. That is, how does cannabis intoxication affect witness statements about criminal events (recall) and identification performance on lineup tasks (recognition)? To our knowledge, only one previous study has examined the effect of cannabis intoxication in an eyewitness setting. Yuille, Tollestrup, Marxsen, Porter, and Herve (1998) randomly assigned Canadian male volunteers to smoke either a marijuana cigarette (*N* = 25) or a placebo cigarette (*N* = 27) in the laboratory. Approximately 5 min later, participants witnessed a staged event. Immediately afterwards, participants were interviewed about it using a free narrative followed by open questions. One week later, they returned to the lab for a second interview and viewed a target‐present or target‐absent lineup. Immediately following the event, participants who had smoked marijuana reported significantly less information about it than did participants in the placebo condition (with no difference in accuracy). One week later, however, no differences between conditions were observed in either recall or lineup identification performance.

The impairments in immediate event recall observed by Yuille et al. (1998) may or may not be relevant in legal settings, depending on what kind of information is forgotten. If cannabis intoxication impairs recall of central details of the witnessed event (e.g., about perpetrators or weapons), that would be more problematic than if it impairs recall of peripheral details (e.g., about the surroundings). In the present study, we therefore distinguished between reported details pertaining to persons, actions, objects, and surroundings in the criminal event (for comparable coding procedures, see, e.g., Milne & Bull, 2002; Vredeveldt, Tredoux, Kempen, & Nortje, 2015). To our knowledge, no previous studies have examined this question. Because we did not have theoretical reasons to think that cannabis would have a larger effect on recall of some types of details than others, this analysis was exploratory in nature.

When an eyewitness makes an identification from a lineup, his self‐reported level of certainty can have considerable consequences for subsequent legal decision making. Jurors as well as legal professionals are much more convinced of the suspect's guilt if the eyewitness indicates that he is very confident in his identification (e.g., Brewer & Burke, 2002; Brigham & Wolfskeil, 1983). It is therefore important to know whether cannabis intoxication affects witnesses' confidence in identification decisions. We propose two tentative possibilities as to how cannabis might affect eyewitness confidence. The first possibility is that witnesses anticipate that smoking cannabis will result in poorer performance on the identification test. Previous research shows that expectations of poor performance can lead to compensatory behaviors (e.g., the hypervigilance effect observed in placebo conditions in alcohol studies; see Testa et al., 2006). Witnesses in the present study who have smoked cannabis might compensate for their perceived poorer performance by adjusting their confidence ratings downward. If this is the case, we would expect to see reduced confidence in identification decisions among witnesses who smoked cannabis, regardless of accuracy.

The second possibility, based on the finding that cannabis can increase insightfulness (Green, Kavanagh, & Young, 2003), is that cannabis increases witnesses' tendencies to introspect on their own cognitive processes. Because accurate witnesses should, on average, have had stronger recognition experiences than inaccurate witnesses (e.g., Charman & Cahill, 2012), this increased introspective focus would lead accurate intoxicated witnesses to weight their recognition experience more heavily, thus leading them to exhibit higher confidence than sober participants do. Some weak support for this hypothesis is provided by Yuille et al. (1998), who observed a nonsignificant trend for intoxicated participants to be more confident in accurate decisions than sober participants. However, because their sample size per cell was relatively small, we cannot draw strong conclusions from their data. 1From Yuille et al.'s (1998) Table 2 (p. 123), we calculated the following sample sizes and means: Intoxicated participants were more confident in accurate decisions (*N* = 19, *M* = 6.37) than sober participants (*N* = 22, *M* = 5.95) but less confident in inaccurate decisions (*N* = 6, *M* = 5.00) than sober participants (*N* = 5, *M* = 5.20). In the present study, we explored these possibilities by examining the effect of cannabis on confidence–accuracy relations for eyewitness identifications.

In sum, although Yuille et al.'s (1998) pioneering study provides useful insights into the potential effects of cannabis on eyewitness memory, it involved only a small sample of participants and a relatively low dosage of cannabis. Our study expands the literature by investigating the effect of cannabis intoxication on eyewitness memory in a field setting with a greater sample size and naturalistic doses. We approached visitors of coffee shops 2In the Netherlands, the term *coffee shop* is used to refer to an alcohol‐free bar or shop that sells soft drugs, specifically, cannabis products (marijuana and hash). in Amsterdam and compared participants who had just smoked cannabis (experimental group) with participants who had not yet smoked cannabis that day (control group). The groups had comparable demographics and experience with cannabis, allowing us to isolate the effect of intoxication status on eyewitness memory. With the basic memory literature (Ranganathan & D'Souza, 2006) and the findings reported by Yuille et al., we predicted that participants under the influence of cannabis would remember fewer correct details about the witnessed incident than sober participants. Further, we predicted that the decrease in correct recall would be dose dependent (i.e., that participants who had smoked more cannabis would recall less about the event). Based on the basic memory literature, we also expected that intoxicated participants would have more false memories, which would be reflected both in the recall of incorrect details about the event and in a higher percentage of false alarms on the lineup identification task (but see Yuille et al., 1998).

## 2 METHOD

### 2.1 Ethical approval

Approval for the study was obtained from the Ethics Committee for Legal and Criminological Research at the Vrije Universiteit Amsterdam.

### 2.2 Participants

We conducted an a priori power analysis. Unfortunately, only one previous study (Yuille et al., 1998) was comparable in research design and outcome measures with the current study. With their effect size for correct recall (*d* = 1.0), the required sample size to achieve 80% power with an α of .05 would be 16 participants per group. Due to the small sample size in their study, however, it may not be wise to take their large effect size as a basis for the power analysis (see Button et al., 2013, on overestimating effect sizes based on small samples). We therefore decided on a larger yet practically feasible sample size (*N =* 60 per group) that would allow us to detect a medium‐sized effect (*d* = 0.5) at 80% power.

We recruited 120 participants (92 male and 28 female), all visitors of coffee shops in Amsterdam. In an attempt to recruit a roughly equal number of sober and intoxicated participants, half of the participants were recruited while entering the coffee shop (most of whom we expected to be sober) and half while leaving the coffee shop (most of whom we expected to be intoxicated). The minimum age required to participate in our study was 18 years, in line with the legal minimum age for entering a coffee shop in the Netherlands. Participants' ages ranged from 18 to 65, with a mean age of 30.15 years (*SD* = 9.99). Forty‐four participants were Dutch, and the remaining 76 participants were visitors from a wide range of other countries. Dutch participants were approached and interviewed in Dutch and foreign participants in English. All participants had normal or corrected‐to‐normal vision and a good command of the Dutch or English language.

### 2.3 Materials

#### 2.3.1 Mock crime

Participants viewed a 2‐min video depicting a robbery in a convenience store. The video shows a cashier behind the counter and several customers walking around the store. After a while, one customer walks up to the cashier, pulls a gun from his trousers, and tells the cashier to give him all her money. He tells another customer to put his hands up. The cashier hands him a bag and he runs out of the store.

#### 2.3.2 Lineups

**Figure 1:**
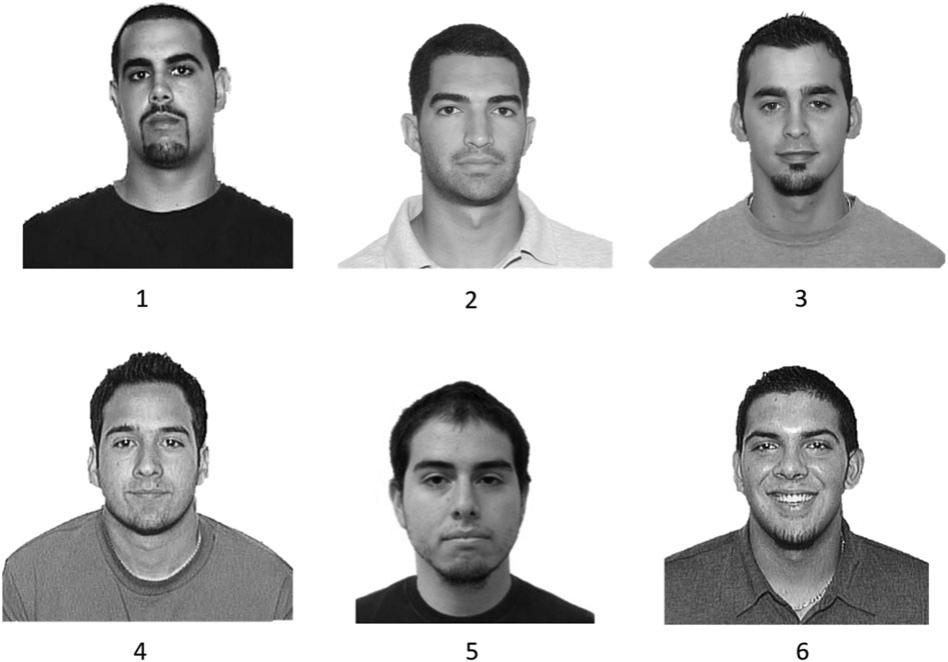
Example target‐present lineup (participants viewed all lineups in color). The target appears in Position 5

Twelve versions of a six‐person photo lineup were constructed, of which six contained a photo of the perpetrator (target‐present) and six a photo of an innocent suspect (target‐absent). The position of the perpetrator in target‐present lineups and of the innocent suspect in target‐absent lineups was systematically varied across the six different positions. Fillers were the same in all lineups and were selected to satisfy two criteria: (a) the lineups were fair according to mock witness tests and (b) the designated innocent suspect was the filler rated most similar to the perpetrator. This latter criterion represents a “worst‐case scenario” in which the innocent suspect happens to resemble the perpetrator the most, which may occur in the real world with some frequency (Tunnicliff & Clark, 2000). A mock witness test (*N* = 35) indicated that the target‐present lineup was fair: Tredoux's (1998) *E*′ = 4.84; 95% CI [3.89, 6.38]. An example lineup is displayed in Figure 1.

### 2.4 Procedure

Participants were recruited while entering or leaving one of four coffee shops in Amsterdam, of which the owners had agreed to cooperate with the research. The data were collected over the course of 15 sessions by the same two researchers, who always went to the coffee shops together. The researchers approached potential participants outside the coffee shop. Once a participant agreed to participate, one researcher sat down with the participant at a table on a terrace in front of the coffee shop to conduct the experiment. All participants took part individually, on a voluntary basis. While conducting the experiment, the researchers were blind to intoxication status, as participants were asked about their cannabis use only at the end of the experiment (notwithstanding the fact that for some participants, it was obvious that they were under the influence). The researchers were not blind to lineup condition, which will be addressed in Section 4.

Participants provided written informed consent and then watched the robbery video on a tablet computer. Next, they completed a 2‐min filler task (a Sudoku) before they were interviewed about the video. Participants were first asked to recall as much as possible about the video (free‐recall phase). Once the participants had told the interviewer everything they could remember, the interviewer asked a predetermined set of open questions about the setting in which the crime took place, the buildup to the crime, the behavior and appearance of the perpetrator, and the other people present in the store (cued‐recall phase). All sessions were audio‐recorded for later transcription.

Upon completion of the interview, participants were told that they would be shown a lineup to attempt an identification. They were informed that the perpetrator may or may not be present, and that they were allowed to respond “don't know.” Participants were randomly assigned to view one of the 12 versions of the lineup (half of which were target‐present and half target‐absent). Participants rated their confidence in their decision on a scale from 0 (*not confident at all*) to 100 (*extremely confident*).

Next, participants provided demographic information (age, gender, native language) and reported how many grams of cannabis they had used that day or, if they did not know how many grams, the number of joints smoked. They provided subjective intoxication ratings by indicating how stoned they felt on a scale of 0 (*not stoned at all*) to 100 (*extremely stoned*). They also indicated whether they had used any other substances that day and, if so, how much of each substance. Finally, participants were debriefed and thanked for their participation.

### 2.5 Data coding

Prior to coding of the data, we created a coding scheme listing details from the robbery video. Details mentioned by participants that were not in the original coding scheme were added progressively. The final coding scheme contained 854 details about the robbery video. Details could be coded as correct (e.g., “the dog was black”), incorrect (e.g., “the dog was brown”), or subjective (e.g., “the dog was cute”). Subjective and repeated details did not count toward the total number of details. Details were also coded as pertaining to persons (e.g., “his hair was black”), actions (e.g., “he stumbled”), objects (e.g., “the gun was black”), or surroundings (e.g., “the store was small”). The coding scheme contained 226 person details, 279 action details, 179 object details, and 170 surrounding details.

All audio‐recorded interviews were transcribed verbatim, and recall performance was coded on the basis of the transcripts. All 120 transcripts were double coded independently by two researchers, blind to condition. Interrater reliability was high for both accuracy (correct, incorrect, subjective), κ = .85, *p* < .001, and type of detail (person, action, object, surrounding), κ = .95, *p* < .001.

## 3 RESULTS

Two participants (one entering and one leaving the coffee shop) who reported having used other drugs (shrooms or cocaine) were excluded from all analyses. Of the remaining 59 participants recruited while entering the coffee shop, 12 had used cannabis that day and 47 had not, according to self‐report. Of the 59 participants leaving the coffee shop, 51 had used cannabis that day and 8 had not. Thus, our final sample consisted of 63 participants under the influence of cannabis and 55 participants not under the influence.

The 63 intoxicated participants all reported that they had smoked joints, which is by far the most common route of cannabis administration in the Netherlands (Van der Pol et al., 2014). For each participant, we recorded the number of grams of cannabis used—when participants indicated the number of joints smoked instead of the number of grams, we converted joints into grams on the basis of an average of 0.3 g of cannabis per joint. 3This amount was based on reports by the owners of participating coffee shops, who stated that their joints contain 0.3 g of cannabis. It is also in line with media reports that joints in Dutch coffee shops contain approximately 0.3 g of cannabis (BNNVARA Woordenboek, n.d.), and with research findings that Dutch frequent cannabis users put an average of 0.28 g of cannabis in a joint (Van der Pol et al., 2013). Participants reported that they had used between 0.06 and 4.00 g of cannabis (equivalent to between one fifth and 12 joints), with a mean reported dose of 0.62 g (*SD* = 0.57). Thus, intoxicated participants had smoked approximately two joints on average. Self‐report ratings of how stoned they felt (on a 0–100 scale) ranged from 0 to 100, with an average of 43.30 (*SD* = 22.00).

Seventy participants were interviewed in their native language (Dutch or English), whereas 48 were not. To check whether this affected the findings, we added native language (yes/no) as a covariate in the analyses of recall performance reported below. 4Running the analyses without the covariate did not change the findings. Prior to analysis, all relevant assumptions were checked, and transformations were applied where necessary, as outlined in the relevant sections. 5Conducting the analyses with the original, untransformed variables did not change the findings. To facilitate interpretation, all descriptive data reported below (means, standard deviations, Cohen's *d*) are of the untransformed variables. All reported *p* values are two‐tailed.

### 3.1 Correct recall

**Figure 2:**
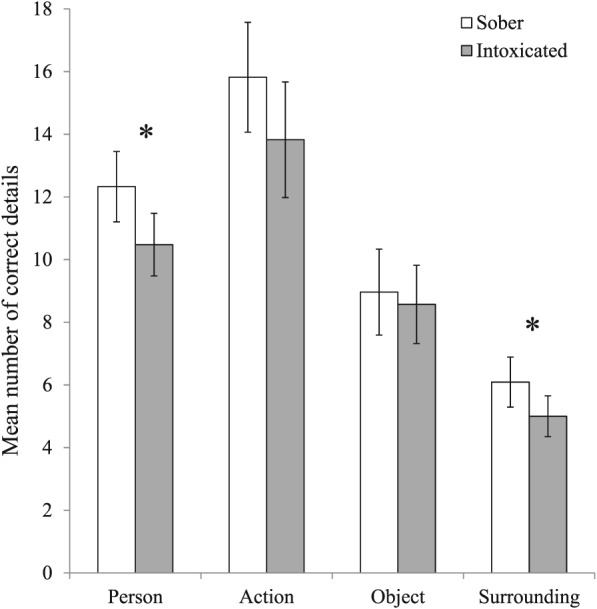
Mean number of correct details about persons, actions, objects, and surroundings reported by sober and intoxicated participants. Asterisks denote significant differences between sober and intoxicated participants (*p* < .05). Error bars represent 95% confidence intervals

We first assessed differences between sober and intoxicated participants in correct recall of different types of details (see Figure 2). To reduce positive skew and leptokurtosis, square‐root transformations were applied prior to analysis. We conducted a 2 (Cannabis: sober, intoxicated) × 4 (Type of Detail: person, action, object, surrounding) multivariate analysis of covariance on the square‐root‐transformed number of correct details, with native language (yes/no) as a covariate. We found a significant effect of native language, *F*(4, 112) = 2.62, *p* = .039, η_p_^2^ = .09: Unsurprisingly, participants who spoke in their native language reported significantly more correct details (*M* = 42.80, *SD* = 14.72) than did participants who spoke in a nonnative language (*M* = 36.85, *SD* = 13.77). After partialling out the effect of language, there was also a significant multivariate effect of cannabis, *F*(4, 112) = 3.53, *p* = .009, η_p_^2^ = .11. As predicted, intoxicated participants reported significantly fewer correct details (*M* = 37.89, *SD* = 14.23) than did sober participants (*M* = 43.24, *SD* = 14.58), *d* = −0.37; 95% CI [−0.74, −0.01]. Simple effects analyses showed that cannabis intoxication significantly reduced the number of correct details reported regarding persons, *F*(1, 115) = 6.42, *p* = .013, η_p_^2^ = .05, and surroundings, *F*(1, 115) = 4.65, *p* = .033, η_p_^2^ = .04, but not actions, *F*(1, 115) = 2.99, *p* = .086, η_p_^2^ = .03, or objects, *F*(1, 115) = 0.02, *p* = .877, η_p_^2^ = .00.

For the intoxicated group, we examined whether the self‐reported dose of cannabis smoked and subjective intoxication ratings were associated with correct recall performance. Because the data for cannabis dose were positively skewed and leptokurtic and could not be transformed into a normal distribution, we used Spearman's correlation for that variable. Contrary to our expectation, we found no significant correlation between the self‐reported amount of cannabis used and the number of correct details reported, *r*_s_ = −.16, *p* = .205. Similarly, there was no significant correlation between subjective intoxication ratings and the number of correct details reported, *r* = −.10, *p* = .421.

### 3.2 Incorrect recall

We then assessed differences between sober and intoxicated participants in incorrect recall of different types of details. Again, square‐root transformations were applied to reduce positive skew and leptokurtosis. A 2 (Cannabis: sober, intoxicated) × 4 (Type of Detail: person, action, object, surrounding) multivariate analysis of covariance on the square‐root‐transformed number of incorrect details, with native language (yes/no) as a covariate, revealed no significant effect of language, *F*(4, 112) = 1.01, *p* = .406, η_p_^2^ = .03, and no significant multivariate effect of cannabis, *F*(4, 112) = 0.93, *p* = .448, η_p_^2^ = .03. Thus, contrary to our hypothesis, intoxicated participants did not report significantly more incorrect details (*M* = 7.17, *SD* = 4.29) than did sober participants (*M* = 6.64, *SD* = 3.61), *d* = 0.14; 95% CI [−0.23, 0.50].

For the intoxicated group, we also examined whether self‐reported cannabis dose was associated with incorrect recall performance, using Spearman's correlation. There was no significant correlation between cannabis dose and the number of incorrect details reported, *r*_s_ = −.02, *p* = .859. Similarly, we found no significant correlation between subjective intoxication ratings and the number of incorrect details reported, *r* = −.17, *p* = .187.

### 3.3 Lineup identification

Table 1 shows the frequency of each type of decision on target‐present and target‐absent lineups, respectively. Because “don't know” responses occurred so infrequently (*N* = 7), they were dropped from all analyses. Cannabis had no significant effect on the distribution of responses (i.e., correct ID, filler ID, and not there) among witnesses who viewed a target‐present lineup, χ^2^(2) = 1.76, *p* = .415, Cramer's *V* = .18. Similarly, cannabis had no significant effect on the distribution of responses (i.e., false ID, filler ID, and not there) among witnesses who viewed a target‐absent lineup, χ^2^(2) = 2.65, *p* = .265, Cramer's *V* = .22. 6Because the false ID rate was low, expected values for two cells were less than 5, violating assumptions of the chi‐square test. However, Fisher's exact test confirmed that the false ID rate was not significantly different between Cannabis conditions, *p* = .216. Thus, cannabis intoxication was not associated with either a decrease in correct identifications or an increase in false alarms. To explore the data further, we collapsed target‐present and target‐absent lineups to assess overall lineup accuracy (i.e., correct vs. incorrect decisions). We found no significant effect of cannabis intoxication on lineup accuracy, χ^2^(1) = 0.97, *p* = .325, Cramer's *V* = .09.

**Table 1:** Number of correct identifications (of the target), false identifications (of the innocent suspect), foil identifications (of another lineup member), no identifications (saying the perpetrator is not present), and “don't know” responses made by sober and intoxicated participants from target‐present and target‐absent lineups

Lineup	Cannabis					
	Sober		Intoxicated		Total	
	*N*	%	*N*	%	*N*	%
Target‐present						
Correct identification	11	36.7	15	50.0	26	43.3
Foil identification	8	26.7	8	26.7	16	26.7
No identification	9	30.0	5	16.7	14	23.3
Don't know	2	6.7	2	6.7	4	6.7
Target‐absent						
False identification	1	4.0	5	15.2	6	10.3
Foil identification	15	60.0	14	42.4	29	50.0
No identification	8	32.0	12	36.4	20	34.5
Don't know	1	4.0	2	6.1	3	5.2

For the intoxicated group, we also examined whether cannabis dose and intoxication ratings could predict accuracy on the lineup task. A logistic regression model with lineup type (target‐present, target‐absent), self‐reported cannabis dose, subjective intoxication ratings, and the various interaction terms as predictors did not explain a significant proportion of the variance in lineup accuracy, Model χ^2^(7) = 3.97, *p* = .783, *R* ^2^ (Hosmer and Lemeshow) = .05. Table 2 displays all statistics regarding this analysis. In sum, we found no evidence for a dose‐dependent effect of cannabis intoxication on the accuracy of decisions on the lineup task.

**Table 2:** Statistics for the logistic regression analysis on lineup identification accuracy, with lineup type (target‐absent or target‐present; Lineup), self‐reported cannabis dose (Dose), subjective intoxication rating (Rating), and the various interaction terms as predictors

Predictors	*b*	*SE*	Wald χ^2^	*df*	*p*	Odds ratio
Lineup	.24	2.12	0.01	1	.910	1.27
Dose	.94	5.05	0.03	1	.853	2.55
Rating	.03	0.07	0.15	1	.702	1.03
Lineup × Dose	−1.33	3.20	0.17	1	.678	0.26
Lineup × Rating	−.03	0.05	0.40	1	.529	0.97
Dose × Rating	−.05	0.11	0.26	1	.607	0.95
Lineup × Dose × Rating	.05	0.07	0.39	1	.531	1.05

### 3.4 Confidence in lineup decisions

**Figure 3:**
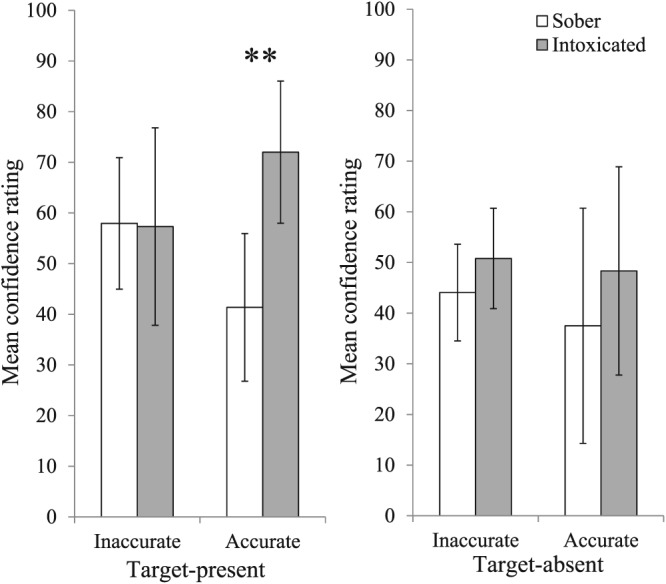
Mean confidence ratings provided by sober and intoxicated participants for accurate and inaccurate decisions from target‐present (left panel) and target‐absent (right panel) lineups. Double asterisk denotes significant difference between sober and intoxicated participants (*p* < .01). Error bars represent 95% confidence intervals

Figure 3 shows confidence in decisions on target‐present and target‐absent lineups, respectively. A 2 (Cannabis: sober, intoxicated) × 2 (Accuracy: accurate, inaccurate) ANOVA on confidence ratings for target‐present lineups revealed no significant effect of accuracy, *F*(1, 52) = 0.02, *p* = .896, η_p_^2^ = .00, but did reveal a significant effect of cannabis, *F*(1, 52) = 4.39, *p* = .041, η_p_^2^ = .08, which was qualified by a significant interaction between cannabis and accuracy, *F*(1, 52) = 4.77, *p* = .034, η_p_^2^ = .08. Simple effects analyses showed that for inaccurate decisions, there was no significant difference between intoxicated and sober participants, *F*(1, 52) = 0.00, *p* = .948, η_p_^2^ = .00. For accurate identifications, however, intoxicated participants were significantly more confident than sober participants, *F*(1, 52) = 8.51, *p* = .005, η_p_^2^ = .14 (see left panel of Figure 3).

A 2 (Cannabis) × 2 (Accuracy) ANOVA on confidence ratings for target‐absent lineups revealed no significant main effects of cannabis, *F*(1, 51) = 1.66, *p* = .204, η_p_^2^ = .03, or accuracy, *F*(1, 51) = 0.44, *p* = .512, η_p_^2^ = .01, and no significant interaction, *F*(1, 51) = 0.09, *p* = .765, η_p_^2^ = .00 (see right panel of Figure 3).

We also examined confidence–accuracy correlations across cannabis conditions. Results were examined separately for nonchoosers and choosers (see Sporer, Penrod, Read, & Cutler, 1995). For nonchoosers, we found no significant association between confidence and accuracy for either sober participants, *r* = −.46, *p* = .066, *N* = 17, or intoxicated participants, *r* = −.10, *p* = .693, *N* = 17. For choosers, we found no significant confidence–accuracy correlation among sober participants, *r* = −.12, *p* = .493, *N* = 35, but a significant correlation among intoxicated participants, *r* = .34, *p* = .028, *N* = 42. The difference between these two correlation coefficients was significant, Fisher's *z* = −1.99, *p* = .047. Thus, intoxicated participants were significantly better than sober participants at judging whether their selection from the lineup was accurate.

## 4 DISCUSSION

Our results revealed three main findings. First, participants who had smoked cannabis reported significantly fewer accurate details when describing the crime than did participants who had not smoked cannabis, with no differences in inaccurate recall. Second, there were no significant differences in lineup identification decisions between sober and intoxicated participants. Third, intoxicated participants were significantly more confident in their accurate identifications than were sober participants, which resulted in a significantly better confidence–accuracy correlation. Each of these findings will be discussed in turn.

### 4.1 Eyewitness recall

Our finding that participants under the influence of cannabis recalled fewer correct details is consistent with the general literature on the effects of cannabis, which has shown that cannabis reduces immediate recall (Ranganathan & D'Souza, 2006). Specifically, it is consistent with the findings reported by Yuille et al. (1998), in which a similar detrimental effect was observed for details recalled about a witnessed event. Note that these results cannot be accounted for by postulating that intoxicated participants were simply less likely to report any information overall (i.e., a criterion shift), as that would have predicted a decrease in inaccurate details, which we did not observe. Instead, we interpret this result as indicating that cannabis adversely affected memory for the witnessed event.

It is not clear from our data at which memory stage the detrimental effect of cannabis occurred. Because participants were under the influence of cannabis both while watching the video and during the interview, we were unable to disentangle the effects of cannabis intoxication on encoding versus retrieval. In basic memory research, findings are mixed on whether cannabis intoxication has a greater impact on encoding or retrieval (see Ranganathan & D'Souza, 2006). In the only previous study on eyewitness memory (Yuille et al., 1998), cannabis‐related impairments observed in immediate event recall were no longer observed in 1‐week delayed recall, when participants had sobered up. In contrast, the literature on state‐dependent memory (e.g., Eich, 1995; Smith, Glenberg, & Bjork, 1978) suggests that an event witnessed while under the influence of cannabis may be best recalled while in the same intoxicated state, whereas an event witnessed while sober may be best recalled sober (see also Darley, Tinklenberg, Roth, & Atkinson, 1974; Goodwin, Powell, Bremer, Hoine, & Stern, 1969; Schreiber Compo et al., 2017). To examine this further, future eyewitness researchers should systematically manipulate cannabis intoxication during encoding and retrieval in a cross‐over design.

To determine how detrimental cannabis intoxication is in legal settings, it is important to know what type of information is affected. We therefore conducted an exploratory analysis to distinguish between details pertaining to persons, actions, objects, and surroundings in the criminal event. This analysis revealed that the decrease in accurate details occurred specifically for person descriptors and surrounding descriptors (and approached significance for action descriptors) but had little effect on object descriptors. Arguably, person descriptors are the most forensically relevant information in a police investigation, as it may lead the police to a suspect or contribute to the evidence incriminating or exonerating a suspect. Surrounding descriptors, in contrast, may be least relevant from a practical perspective. Our findings thus suggest that cannabis intoxication impairs recall of central as well as peripheral information. This analysis was, however, exploratory in nature, and as such we do not wish to overinterpret it. Future studies may wish to further explore the specific types of details for which cannabis intoxication selectively impairs recall.

### 4.2 Lineup identification

We found that sober and intoxicated participants did not differ significantly in terms of lineup identification accuracy. This finding is in line with the only previous study on the effects of cannabis intoxication on eyewitness identifications (Yuille et al., 1998), but their lineup analysis was severely underpowered (*N* = 52). Our replication of this null effect with a greater sample size (*N* = 111) suggests that any potential detrimental effect of cannabis on lineup identification accuracy is relatively small, if it exists at all—at least among the dosages observed in the current study. Further, our results are consistent with other studies that have examined the effects of cannabis on memory more generally, which have found larger detrimental effects of cannabis on recall memory than on recognition memory (Ranganathan & D'Souza, 2006).

In the present study, the researchers who conducted the experiments knew whether participants were viewing a target‐present or target‐absent lineup, and which lineup member was the guilty or innocent suspect, respectively. When lineup administrators know who the suspect is, they can inadvertently influence the witness's decision toward the suspect (Charman & Quiroz, 2016; Greathouse & Kovera, 2009). Legal psychologists therefore recommend double‐blind lineup procedures (e.g., National Academy of Sciences, 2014). In real life, however, it is unfortunately still very common for lineups to be carried out by administrators who know who the suspect is (Rodriguez & Berry, 2013). Thus, although the nonblind administration of lineups in the present study represented nonoptimal conditions, it mirrored common practice in real police investigations.

The level of confidence expressed in a lineup identification can have a significant impact on judgments about the credibility of the eyewitness and the likely guilt of the suspect (e.g., Brewer & Burke, 2002; Brigham & Wolfskeil, 1983). Interestingly, similar to the trend observed by Yuille et al. (1998), we found that participants under the influence of cannabis were significantly more confident in accurate identifications compared to sober participants and significantly better at judging whether their lineup decision was accurate. This finding is not in line with the hypothesis that intoxicated participants would compensate for anticipated poorer performance by adjusting their confidence ratings downward. It is in line, however, with the hypothesis that cannabis leads witnesses to become more focused on their cognitive processes, thus leading accurate witnesses to weight their recognition experiences particularly heavily and increase their confidence. This effect could be due to the pharmacological properties of cannabis itself (i.e., the drug increases internal focus), or due to expectancies regarding the drug (i.e., in anticipation of poor performance, witnesses may pay more attention to their internal cues). Future research is necessary to test the replicability of this finding and, if it replicates, to differentiate between these hypotheses.

### 4.3 Limitations

Some limitations of the present study have already been noted, but perhaps the most important limitation was the lack of control associated with the nature of the field study. Due to ethical reasons, we were unable to randomly assign participants on the street to smoke cannabis or not. We attempted to quasi‐randomly assign participants by approaching them as they either entered a coffee shop (the majority of whom we assumed would be sober) or exited the coffee shop (the majority of whom we assumed would be intoxicated). Although this method was largely successful (80% of participants entering the coffee shop were sober; 86% of people exiting the coffee shop were intoxicated), it was an imperfect proxy for intoxication, necessitating a regrouping of about 17% of participants for data analysis purposes. In a similar vein, we could not establish with certainty the precise amount of cannabis smoked, nor the exact amount of time that had passed since smoking cannabis. Ideally, studies examining the effects of cannabis should randomly assign participants to intoxication condition and should standardize cannabis dose and delay. On the other hand, such an increase in control is almost inevitably accompanied by a trade‐off in ecological validity. A strength of this study is that we tested participants who had chosen to smoke cannabis without intervention from the experimenters, in an environment in which cannabis is usually smoked. As such, our sample was probably more representative of the typical eyewitness under the influence of cannabis than participant samples used in previous laboratory research (e.g., Yuille et al., 1998).

## 5 CONCLUSION

Despite the frequency with which people use cannabis, there is almost no research examining its effects on eyewitness memory. Our observed results are consistent with the general cannabis literature: Intoxicated people (a) provided less accurate information during a recall task, an effect that seems to reflect poorer memory on the part of the intoxicated witnesses, but (b) showed no impairment on a recognition task (i.e., making identification decisions from a lineup). From a practical perspective, more research is needed to determine whether the loss of accurate information reported can be recovered by having witnesses sober up. Fortunately, results suggest that the detrimental effects of cannabis on recall memory are not mirrored by detrimental effects on a lineup task.

## References

[acp3414-bib-0001] Balota, D. A. , Cortese, M. J. , Duchek, J. M. , Adams, D. , Roediger, H. L. , McDermott, K. B. , & Yerys, B. E. (1999). Veridical and false memories in healthy older adults and in dementia of the Alzheimer's type. Cognitive Neuropsychology, 16, 361–384. 10.1080/026432999380834

[acp3414-bib-0002] BNNVARA Woordenboek . (n.d.). Joint. Retrieved November 23, 2017 from https://spuitenenslikken.bnnvara.nl/woordenboek/item/joint

[acp3414-bib-0003] Brainerd, C. J. , & Reyna, V. F. (2002). Fuzzy‐trace theory and false memory. Current Directions in Psychological Science, 11, 164–169. 10.1111/1467-8721.00192

[acp3414-bib-0004] Brewer, N. , & Burke, A. (2002). Effects of testimonial inconsistencies and eyewitness confidence on mock‐juror judgments. Law and Human Behavior, 26, 353–364. 10.1023/a:1015380522722 12061623

[acp3414-bib-0005] Brigham, J. C. , & Wolfskeil, M. P. (1983). Opinions of attorneys and law enforcement personnel on the accuracy of eyewitness identification. Law and Human Behavior, 7, 337–349. 10.1007/BF01044736

[acp3414-bib-0006] Button, K. S. , Ioannidis, J. P. A. , Mokrysz, C. , Nosek, B. A. , Flint, J. , Robinson, E. S. J. , & Munafo, M. R. (2013). Power failure: Why small sample size undermines the reliability of neuroscience. Nature Reviews Neuroscience, 14, 365–376. 10.1038/nrn3475 23571845

[acp3414-bib-0103] Charman, S. D. , & Cahill, B. S. (2012). Witnesses' memories for lineup fillers postdicts their identification accuracy. Journal of Applied Research in Memory and Cognition, 1, 11–17. 10.1016/j.jarmac.2011.08.00

[acp3414-bib-0007] Charman, S. D. , & Quiroz, V. (2016). Blind sequential lineup administration reduces both false identifications and confidence in those false identifications. Law and Human Behavior. Advance Online Publication. doi:10.1037/lhb0000197 27227276

[acp3414-bib-0008] Crossland, D. , Kneller, W. , & Wilcock, R. (2016). Intoxicated witnesses: Testing the validity of the alcohol myopia theory. Applied Cognitive Psychology, 30, 270–281. 10.1002/acp.3209

[acp3414-bib-0009] Darley, C. F. , Tinklenberg, J. R. , Roth, W. T. , & Atkinson, R. C. (1974). The nature of storage deficits and state‐dependent retrieval under marihuana. Psychopharmacology, 37, 139–149. 10.1007/bf00437420 4844119

[acp3414-bib-0010] D'Souza, D. C. , Perry, E. , MacDougall, L. , Ammerman, Y. , Cooper, T. , Wu, Y. T. , … H, J. (2004). The psychotomimetic effects of intravenous delta‐9‐tetrahydrocannabinol in healthy individuals: Implications for psychosis. Neuropsychopharmacology, 29, 1558–1572. 10.1038/sj.npp.1300496 15173844

[acp3414-bib-0011] Eich, J. E. (1995). Mood as a mediator of place dependent memory. Journal of Experimental Psychology: General, 124, 293–308. 10.1037/0096-3445.124.3.293 7673863

[acp3414-bib-0012] Evans, J. R. , Compo, N. S. , & Russano, M. B. (2009). Intoxicated witnesses and suspects: Procedures and prevalence according to law enforcement. Psychology, Public Policy, and Law, 15, 194–221.

[acp3414-bib-0013] Goodwin, D. W. , Powell, B. , Bremer, D. , Hoine, H. , & Stern, J. (1969). Alcohol and recall: State‐dependent effects in man. Science, 163, 1358–1360. 10.1126/science.163.3873.1358 5774177

[acp3414-bib-0014] Greathouse, S. M. , & Kovera, M. B. (2009). Instruction bias and lineup presentation moderate the effects of administrator knowledge on eyewitness identification. Law and Human Behavior, 33, 70–82. 10.1007/s10979-008-9136-x 18594956

[acp3414-bib-0015] Green, B. , Kavanagh, D. , & Young, R. (2003). Being stoned: A review of self‐reported cannabis effects. Drug & Alcohol Review, 22, 453–460. 10.1080/09595230310001613976 14660135

[acp3414-bib-0016] Hagsand, A. V. , Roos af Hjelmsäter, E. , Granhag, P. A. , Fahlke, C. , & Söderpalm Gordh, A. (2017). Witnesses stumbling down memory lane: The effects of alcohol intoxication, retention interval, and repeated interviewing. Memory, 25, 531–543. 10.1080/09658211.2016.1191652 27249626

[acp3414-bib-0017] Hart, C. L. , Ilan, A. B. , Gevins, A. , Gunderson, E. W. , Role, K. , Colley, J. , & Foltin, R. W. (2010). Neurophysiological and cognitive effects of smoked marijuana in frequent users. Pharmacology Biochemistry and Behavior, 96, 333–341. 10.1016/j.pbb.2010.06.003 PMC5858872 20600251

[acp3414-bib-0018] Heishman, S. J. , Arasteh, K. , & Stitzer, M. L. (1997). Comparative effects of alcohol and marijuana on mood, memory, and performance. Pharmacology Biochemistry and Behavior, 58, 93–101. 10.1016/S0091-3057(96)00456-X 9264076

[acp3414-bib-0019] Ilan, A. B. , Smith, M. E. , & Gevins, A. (2004). Effects of marijuana on neurophysiological signals of working and episodic memory. Psychopharmacology, 176, 214–222. 10.1007/s00213-004-1868-9 15502936 PMC1463999

[acp3414-bib-0020] La Rooy, D. , Nicol, A. , & Terry, P. (2013). Intoxicated eyewitnesses: The effects of alcohol on eyewitness recall across repeated interviews. Open Journal of Medical Psychology, 2, 8 10.4236/ojmp.2013.23017

[acp3414-bib-0021] Loef, L. , Heijke, M. , & Van Dijk, B. (2010). Typologie van plegers van geweldsdelicten. Amsterdam: DSP groep.

[acp3414-bib-0022] Miller, L. L. , & Cornett, T. L. (1978). Marijuana: Dose effects on pulse rate, subjective estimates of intoxication, free recall and recognition memory. Pharmacology Biochemistry and Behavior, 9, 573–577. 10.1016/0091-3057(78)90205-8 733845

[acp3414-bib-0023] Miller, L. L. , Cornett, T. L. , & Wikler, A. (1979). Marijuana: Effects on pulse rate, subjective estimates of intoxication and multiple measures of memory. Life Sciences, 25, 1325–1330. 10.1016/0024-3205(79)90398-9 513962

[acp3414-bib-0024] Miller, L. L. , McFarland, D. , Cornett, T. L. , & Brightwell, D. (1977). Marijuana and memory impairment: Effect on free recall and recognition memory. Pharmacology Biochemistry and Behavior, 7, 99–103. 10.1016/0091-3057(77)90191-5 918141

[acp3414-bib-0025] Milne, R. , & Bull, R. (2002). Back to basics: A componential analysis of the original cognitive interview mnemonics with three age groups. Applied Cognitive Psychology, 16, 743–753. 10.1002/acp.825

[acp3414-bib-0026] National Academy of Sciences (2014). Identifying the culprit: Assessing eyewitness identification. Washington, DC: The National Academies Press.

[acp3414-bib-0027] Palmer, F. , Flowe, H. D. , Takarangi, M. K. T. , & Humphries, J. E. (2013). Intoxicated witnesses and suspects: An archival analysis of their involvement in criminal case processing. Law and Human Behavior, 37, 54–59. 10.1037/lhb0000010 23025346

[acp3414-bib-0028] Ranganathan, M. , & D'Souza, D. C. (2006). The acute effects of cannabinoids on memory in humans: A review. Psychopharmacology, 188, 425–444. 10.1007/s00213-006-0508-y 17019571

[acp3414-bib-0029] Riba, J. , Valle, M. , Sampedro, F. , Rodriguez‐Pujadas, A. , Martinez‐Horta, S. , Kulisevsky, J. , & Rodriguez‐Fornells, A. (2015). Telling true from false: Cannabis users show increased susceptibility to false memories. Molecular Psychiatry, 20, 772–777. 10.1038/mp.2015.36 25824306 PMC4441258

[acp3414-bib-0030] Rodriguez, D. N. , & Berry, M. A. (2013). Eyewitness science and the call for double‐blind lineup administration. Journal of Criminology, 2013, 1–10. 10.1155/2013/530523

[acp3414-bib-0031] Schreiber Compo, N. , Carol, R. N. , Evans, J. R. , Pimentel, P. , Holness, H. , Nichols‐Lopez, K. , … Furton , K. G . (2017). Witness memory and alcohol: The effects of state‐dependent recall. Law and Human Behavior *,* 41, 202–215. doi:10.1037/lhb0000224 27786509

[acp3414-bib-0032] Smith, S. M. , Glenberg, A. M. , & Bjork, R. (1978). Environmental context and human memory. Memory & Cognition, 6, 342–353. 10.3758/bf03197465

[acp3414-bib-0033] Sporer, S. L. , Penrod, S. D. , Read, D. , & Cutler, B. L. (1995). Choosing, confidence, and accuracy: A meta‐analysis of the confidence–accuracy relation in eyewitness identification studies. Psychological Bulletin, 118, 315–327. 10.1037/0033-2909.118.3.315

[acp3414-bib-0034] Testa, M. , Fillmore, M. T. , Norris, J. , Abbey, A. , Curtin, J. J. , Leonard, K. E. , … Hayman , L. W . (2006). Understanding alcohol expectancy effects: Revisiting the placebo condition. Alcoholism: Clinical and Experimental Research *,* 30, 339–348. doi:10.1111/j.1530-0277.2006.00039.x PMC1403295 16441283

[acp3414-bib-0035] Tredoux, C. G. (1998). Statistical inference on lineup measures. Law and Human Behavior, 22, 217–237. 10.1023/a:1025746220886

[acp3414-bib-0036] Tunnicliff, J. L. , & Clark, S. E. (2000). Selecting foils for identification lineups: Matching suspects or descriptions? Law and Human Behavior, 24, 231–258. 10.1023/A:1005463020252 10810840

[acp3414-bib-0037] Van der Pol, P. , Liebregts, N. , Brunt, T. , Van Amsterdam, J. , De Graaf, R. , Korf, D. J. , … Van Laar, M. (2014). Cross‐sectional and prospective relation of cannabis potency, dosing and smoking behaviour with cannabis dependence: An ecological study. Addiction, 109, 1101–1109. 10.1111/add.12508 24628797

[acp3414-bib-0038] Van der Pol, P. , Liebregts, N. , De Graaf, R. , Korf, D. J. , Van den Brink, W. , & Van Laar, M. (2013). Validation of self‐reported cannabis dose and potency: An ecological study. Addiction, 108, 1801–1808. 10.1111/add.12226 23627816

[acp3414-bib-0039] Vredeveldt, A. , Tredoux, C. G. , Kempen, K. , & Nortje, A. (2015). Eye remember what happened: Eye‐closure improves recall of events but not face recognition. Applied Cognitive Psychology, 29, 169–180. 10.1002/acp.3092

[acp3414-bib-0040] WODC, & Trimbos Instituut . (2016). Nationale drug monitor: Jaarbericht 2016. Retrieved from https://assets.trimbos.nl/docs/24dd30ba-464f-4dcd-a740-20ac058d310b.pdf.

[acp3414-bib-0041] Yuille, J. C. , Tollestrup, P. A. , Marxsen, D. , Porter, S. , & Herve, H. F. (1998). An exploration on the effects of marijuana on eyewitness memory. International Journal of Law and Psychiatry, 21, 117–128. 10.1016/S0160-2527(97)00027-7 9526721

